# High-level ciprofloxacin resistance in Neisseria gonorrhoeae: first report from Israel.

**DOI:** 10.3201/eid0701.010126

**Published:** 2001

**Authors:** M Dan, F Poch, D Shpitz, B Sheinberg


### High-Level Ciprofloxacin Resistance in *Neisseria gonorrhoeae*: First Report from Israel

**To the Editor:** We report a case of male gonococcal urethritis that persisted despite ciprofloxacin therapy. The isolate was found to be highly resistant (MIC 32 µg/mL).

A 30-year-old man visited his family physician with a 2-day history of urethral discharge and dysuria. The symptoms began 7 days after a single, unprotected orogenital contact with a female hitchhiker. The patient denied exchange of money for the act and reported no other recent sex partners or travel outside Israel. After a urethral swab was obtained for culture, the patient received a single dose of ciprofloxacin (500 mg orally). Growth of *Neisseria gonorrhoeae* was subsequently reported. However, symptoms persisted, and a regimen of doxycycline (100 mg orally twice a day for 10 days) was initiated. After temporary clinical improvement, the patient returned with worsening symptoms: bloody urethral discharge, severe dysuria, edema of the penis, and painful erection. *N. gonorrhoeae* was reisolated from a repeat urethral swab. When a single dose of ceftriaxone (250 mg) was administered intramuscularly, clinical cure was prompt.

Susceptibility testing was performed on the second isolate by using the E-test method (AB Biodisk, Solna, Sweden) on a medium containing GC agar base and 1% defined growth supplement. The MIC of ciprofloxacin was 32 µg/mL, penicillin 1.5 µg/mL, tetracycline 2 µg/mL, and ceftriaxone 0.016 µg/mL. The isolate did not produce beta-lactamase. It was classified as a CMRNG$^{PT}$ phenotype (*N. gonorrhoeae* with chronomosomally mediated resistance to both penicillin and tetracycline).

Gonorrhea was considered a rare disease in Israel in the 1990s: the average annual incidence was 0.89 reported cases per 100,000 population (1). Most laboratories did not carry appropriate media, and susceptibility testing of *N. gonorrhoeae* was not performed routinely. Quinolones and spectinomycin are the antibiotics most commonly used to treat the infection. Nevertheless, we are not aware of any instance of clinical failure following fluoroquinolone therapy. More recently, however, the incidence of gonorrhea has been increasing (2). In response, a surveillance program for monitoring antimicrobial resistance in *N. gonorrhoeae* has been launched.

Fluoroquinolones and cephalosporins became the recommended drugs for treatment of gonococcal infection after penicillin- and tetracycline-resistant *N. gonorrhoeae* appeared (3). Gonococcal strains with reduced in vitro susceptibility to fluoroquinolones (MIC, 0.125 µg/mL to 0.5 µg/mL) were first described in the mid-1980s (4) and are now occurring worldwide (5).

Fluoroquinolone-resistant *N. gonorrhoeae* (ciprofloxacin MIC ≥1.0 µg/mL) emerged during the 1990s and became well established in several Asian countries (6). In Japan, the rate of ciprofloxacin resistance increased from 6.6% in 1993-1994 to 24.4% in 1997-1998 (7). More recently, high-level resistance to ciprofloxacin and reports of treatment failure have appeared (8). Strains with ciprofloxacin MICs of ≥8.0 µg/mL were first isolated in 1994 (6) and are detected mostly in the Far East. Two cases of gonococcal infection by strains with an MIC of 16 µg/mL were recently reported in the United States (9). Gonococcal resistance to fluoroquinolones is associated with mutations in the genes encoding DNA gyrase (gyrA) and topoisomerase (parC) as well as change in porin permeability and reduced intracellular drug accumulation (6). In view of the increasing resistance to fluoroquinolones, ceftriaxone, cefixime, or spectinomycin is now recommended if an infection was acquired in Asia or other areas with known fluoroquinolone resistance (9).

Our patient reported no travel to the Far East, and his sex partner, who could not be located for follow-up, was not Asian; her travel history was unknown. The mode of transmission of this infection was fellatio. Condoms are often not used in this form of intercourse, even by those who regularly use condoms for genito-genital sex, because of the mistaken belief that infection is not spread through this form of intercourse. It is now well established that oral sex plays an important role in HIV transmission (10), and condoms should be used with any form of intimate sexual contact.

**Michael Dan,* Francesca Poch,* Daniel Shpitz,† and Bracha Sheinberg†**
*Infectious Disease Unit, E. Wolfson Hospital, Holon, Israel, and †Maccabi Health Services, Rishon-le-Zion, Israel

### References

1. The Israel Center for Diseases Control. Notifiable infectious disease in Israel, 1951-1995. Israel: State of Israel: Ministry of Health; 1996. pub. no. 201.
2. District Health Office, Tel-Aviv. Report on emerging issues, July-December 1999. State of Israel: Ministry of Health; Jan 10, 2000.
3. Centers for Disease Control and Prevention. Guidelines for treatment of sexually transmitted diseases. Mor Mortal Wkly Rep 1998;47(RR-1):1-116.
4. Tapsall JW, Shultz TR, Phillips EA. Characteristics of *Neisseria gonorrhoeae* isolated in Australia showing decreased sensitivity to quinolone antibiotics. Pathology 1992;24:27-31.
5. Quinn TC. The impact of antimicrobial resistance on the treatment of sexually transmitted diseases. Infect Dis Clin North Am 1997;11:884-904.
6. Knapp JS, Fox KK, Trees DL, Whittington WL. Fluoroquinolone resistance in *Neisseria gonorrhoeae*. Emerg Infect Dis 1997;3:33-8.
7. Tanaka M, Nakayama H, Haraoka M, Saika T, Kobayashi I, Naito S. Antimicrobial resistance of *Neisseria gonorrhoeae* and high prevalence of ciprofloxacin-resistant isolates in Japan, 1993 to 1998. J Clin Microbiol 2000;38:521-5.
8. Tapsall JW, Limnios EA, Thacker C, Donovan B, Lynch SD, Kirky LJ, et al. High-level quinolone resistance in *Neisseria gonorrhoeae*: a report of two cases. Sex Transm Dis 1995;22:310-11.
9. Centers for Disease Control and Prevention. Fluoroquinolone-resistant *Neisseria gonorrhoeae*–San Diego, California, 1997. Mor Mortal Wkly Rep 1998;47:405-8.
10. Dillon B, Hecht FM, Swanson M, Goupil-Sormany I, Grant RH, Chesney MA, et al. Primary HIV infections associated with oral transmission. Presented at the 7th Conference on Retroviruses and Opportunistic Infections, San Francisco, Jan 30–Feb 2, 2000 (Abstract 473).

### An Unusual Bacterium Causing a Brain Abscess

**To the Editor:** Intracranial abscesses are an important cause of illness and death in a neurologic/neurosurgical unit. Early presumptive clinical diagnosis supported by radiologic evidence (computerized axial tomography [CAT] scan and magnetic resonance imaging) is the mainstay of diagnosis (1). Abscess contents are aspirated under stereotaxic guidance and cultured to isolate causative organisms and determine their antibiotic sensitivities. Organisms isolated from brain abscesses are usually streptococci, anaerobic and facultative gram-negative bacilli, staphylococci, or pseudomonads (2).

A 24-year-old male farmer came to us with progressive headache, dizziness, and a low-grade fever of 2 weeks' duration. He had had a pimple on his right cheek approximately 3 weeks before, which had discharged "bluish" pus on forcible evacuation and subsequently healed without treatment. No focal neurologic signs were detected on physical examination. Because an intracranial space-occupying lesion was suspected, a lumbar puncture was withheld. Later, a CAT scan of the patient's head revealed a right-sided temporoparietal space-occupying lesion approximately 3 cm in diameter, suggestive of a unilocular brain abscess. The abscess was needle aspirated under stereotaxic guidance, and the pus was cultured aerobically and anaerobically. After 24 hours of aerobic incubation on MacConkey agar at 37°C, a pure growth of violet-colored colonies appeared, identified as *Chromobacterium violaceum* by the 20E API system (Biomerieux, France).

Other initial laboratory findings were as follows: blood leukocyte count, 16,200 cells/µL (84% neutrophils, 15% lymphocytes, 1% eosinophils); erythrocyte sedimentation rate (Westergren method), 22 mm/hour; C-reactive protein concentration, 96 mg/L; and fasting blood sugar concentration, 5.1 mmol/L. Blood urea and C-reactive protein concentrations after 3 weeks of antibiotic treatment were 4.6 mmol/L and <6 mg/L, respectively.

The organism was sensitive to imipenem and ciprofloxacin and resistant to cefotaxime and ceftriaxone, by the Stokes comparative disk-diffusion antibiotic sensitivity testing method (3). Ciprofloxacin (as lactate) was administered intravenously, 400 mg twice a day, for 4 weeks. Repeated CAT scans, clinical symptoms, and serial C-reactive protein levels indicated rapid regression of the abscess followed by complete cure.

*C. violaceum* is a gram-negative bacillus present in soil and aquatic environments of tropical and subtropical countries or regions such as Trinidad, Guyana, India, Malaysia, Florida, and South Carolina. It is a bacterium of low virulence, occasionally causing skin

